# Caffeic acid phenethyl ester (CAPE) confers wild type p53 function in p53^Y220C^ mutant: bioinformatics and experimental evidence

**DOI:** 10.1007/s12672-021-00461-2

**Published:** 2021-12-20

**Authors:** Navaneethan Radhakrishnan, Jaspreet Kaur Dhanjal, Anissa Nofita Sari, Yoshiyuki Ishida, Keiji Terao, Sunil C. Kaul, Durai Sundar, Renu Wadhwa

**Affiliations:** 1 DAILAB, Department of Biochemical Engineering & Biotechnology, Indian Institute of Technology (IIT) Delhi, Hauz Khas, New Delhi, 110 016 India; 2 AIST-INDIA DAILAB, DBT-AIST International Center for Translational & Environmental Research (DAICENTER), National Institute of Advanced Industrial Science & Technology (AIST), Tsukuba, 305 8565 Japan; 3 Department of Computational Biology, Indraprastha Institute of Information Technology Delhi, Okhla Industrial Estate, Phase III, New Delhi, 110 020 India; 4 Cyclochem Co., Ltd., 7-4-5 Minatojima-minamimachi, Chuo-ku, Kobe, 650 0047 Japan

**Keywords:** p53^Y220C^, CAPE, p53^wt^, Restoration, Anticancer, Therapy

## Abstract

Mutations in the tumor suppressor protein p53 is a prevalent feature in majority of cancers resulting in inactivation of its activities related to control of cell cycle progression and proliferation. p53^Y220C^ is one of the common hotspot mutations that causes decrease in its thermodynamic stability. Some small molecules have been shown to bind to the mutated site and restore its wild type thermodynamics and tumor suppressor function. In this study, we have explored the potential of caffeic acid phenethyl ester (CAPE—a bioactive compound from propolis) to interact with p53^Y220C^ and restore its wild type p53 (p53^wt^) transcription activation and tumor suppressor activities. We recruited computational methods, viz. molecular docking, molecular dynamics simulations and free energy calculations to study the interaction of CAPE at the mutation crevice and found that it has potential to restore p53^wt^ function of the p53^Y220C^ mutant similar to a previously described restoration molecule PK7242. We provide cell-based experimental evidence to these predictions and suggest CAPE as a potential natural drug for treatment of p53^Y220C^ mutant harboring cancers.

**Supplementary Information:**

The online version contains supplementary material available at 10.1007/s12672-021-00461-2.

## Introduction

Cancer is a complex disorder. Commonly defined as a disease of proliferation, it is an outcome of large number of molecular changes in cells yielding activation of oncogenes and/or inactivation of tumor suppressor genes and involves intricate network of interactions between the tumor and host tissue. Among the various genetic aberrations leading to the onset and progression of carcinogenesis, functional inactivation of p53 tumor suppressor protein (by mutations or other mechanisms) has been found in almost every type of cancer. However, the rate of mutagenesis varies greatly—from 10% in hematopoietic malignancies to almost 100% in high-grade serous carcinoma [1]. p53, also known as the guardian of genome, plays key role in tumor suppression by inducing growth arrest, apoptosis and senescence [2–4]. Wild type p53 activities, besides proliferation, have also been shown to inhibit cancer metastasis [5], the process of angiogenesis [6] and sensitize the cancer cells to chemotherapeutic agents [7]. Advances in high-throughput sequencing of cancer genomes have led to the identification of p53 mutational signatures with clinical significance. Based on various crystallographic studies on p53 mutants, these can mainly be classified in three categories—(i) contact mutations occurring in the DNA-binding region of p53 protein (like mutations involving amino acid residues R273 and R248), (ii) structural mutations causing conformational instability in p53 protein (like mutations involving amino acid residues R175, G245, R249 and R282), and (iii) oncomorphic mutations leading to the loss of wild type p53 function whilst the gain of oncogenic properties (mutations involving residues R248, R273 and R175). Therefore, mutations in p53 may inactivate or differently activate the molecule. p53^Y220C^ is one of the frequently observed mutant forms of p53 in cancer cells [8]. p53^Y220C^ mutant contains a unique surface crevice, formed due to the replacement of tyrosine with cysteine at 220th amino acid residue position [9]. The solvation of this crevice decreases the thermodynamical stability and hence the activity of the protein [10, 11]. It has been reported that this surface crevice is druggable, and hence the mutant can be rescued by small molecules that can bind to the crevice and stabilize the molecule [9, 12]. As this crevice is distant from the DNA-binding interface or any other protein–protein interaction interfaces of p53, small molecules can interact with the mutation crevice without interfering with binding of the protein to its interacting partners [12, 13]. Different synthetic molecules like PhiKan 083, PK7088, PK7242 and MB725 have been reported to cause reactivation of the mutant by binding to the mutation crevice [9, 12, 13]. Since introduction of wild type p53 activity in mutant p53 harboring cancer cells causes their growth arrest/apoptosis, the anticancer therapies targeting mutant p53/ activation of wild type p53 function have been considered as a valid and effective drug discovery approach. Not only small molecules, other approaches like introduction of mutation at secondary site [14], use of antibodies [15, 16] or short peptides [17, 18] have also been reported to restore DNA binding in the mutant p53 protein resulting in activation of its transcriptional and tumor suppressor functions. A previous study from our group has also shown the potential of withanolides, withaferin-A and withanone, in restoring the wild type p53 activity in p53^Y220C^ mutant harboring cancer cells [19].

Caffeic acid phenethyl ester (CAPE) is a bioactive compound extracted from honeybee propolis. Recent studies have shown that CAPE possesses anticancer activity against a variety of cancer types and is more toxic to cancer in comparison to healthy cells [20–22]. The potential anticancer activity of CAPE has been attributed to various mechanism of its action that involve—(i) inhibition of NF-kappa B, iNOS signaling, PAK1 and histone deacetylase [23–27], (ii) restoration of gap junctions and downregulation of p21ras [28, 29] (iii) induction of apoptosis by regulation of p53, Bax and Bak [30–32], (iv) targeting VEGF mediated processes like angiogenesis, invasion and metastasis [33, 34], (v) controlling epithelial–mesenchymal transition by modulating Vimentin and Twist 2 [12], (vi) downregulation of Akt signaling pathway [35–37], and (vii) abrogation of mortalin-p53 interactions causing nuclear translocation and reactivation of the transcriptional activation function of p53 resulting in growth arrest of cancer cells [38]. Further, it has also been shown to inhibit cell migration, sensitize cancer cells to other chemotherapeutic agents [39–41] and act as radioprotector and radiosensitizer [42].

We had earlier reported that CAPE causes downregulation of mortalin and also blocks its interaction with p53, resulting in reactivation of its transcriptional activation function [38, 43]. CAPE-treated cells showed nuclear translocation and enrichment of wild type p53. This phenomenon was accompanied by activation of wild type p53 transcriptional activation function, upregulation of p21^WAFI^ and growth arrest in cancer cells [38, 43]. In the present study, we investigated its effect on p53^Y220C^ mutant. Molecular modelling methods of docking and molecular dynamics simulations were used to examine the interaction of CAPE with p53^Y220C^, assuming that CAPE could cause reactivation of p53^Y220C^ by binding to its mutation crevice like some other reported small molecules including aminobenzothiazole derivatives and Ashwagandha withanolides, withaferin-A and withanone [9, 12, 13, 19]. Bioinformatics and experimental analyses revealed that CAPE could confer wild type p53 function to p53^Y220C^ yielding growth arrest/apoptosis in cancer cells.

## Materials and methods

### Molecular structure of proteins and ligand

The three-dimensional structures of the proteins were downloaded from Protein Data Bank (www.rcsb.org). The PDB identifiers of the p53 structures are 3ZME and 1UOL [44, 45]. 3ZME is the p53^Y220C^ mutant with the stabilizing molecule PK7242 bound to its mutation crevice. 3ZME was used for analyzing the interaction of CAPE in the mutation crevice with reference to that of the bound PK7242. 1UOL is a stable-variant of p53 structurally and functionally similar to wild-type. 1UOL was used as a wild-type representative for comparison with the equilibrated structures of CAPE-bound p53^Y220C^ and PK7242-bound p53^Y220C^. The three-dimensional structures of CAPE and PK7242 were downloaded from PubChem database (CID: 5281787 and CID: 91885429 respectively). The proteins were prepared using Schrodinger Maestro Suite 2020 [46]. Hydrogens were added and bond orders were assigned in the protein structure. Ionization state ‘2^+^’ was assigned for the zinc atom that was present in both the crystal structures of p53. Missing side chains of the residues were added. N and C termini were capped using *N*-acetyl and *N*-methyl amide groups respectively. PROPKA was used to assign the protonation states corresponding to pH of 7 [46]. The ionization states of ligands at target pH of 7 were generated and optimized using LigPrep module in Maestro [46].

### Generation of protein-drug complexes by molecular docking

The Glide module in Maestro was used for the docking of CAPE and PK7242 to the mutation crevice of p53^Y220C^ [46]. Docking grid of dimensions 10 Å on each side was generated around residue 220 of p53^Y220C^ and the ligands were docked using the Extra-precision (XP) docking and scoring method in Glide [46]. Flexible ligand docking was used, and the top scoring docking poses were chosen for further analysis.

### Molecular dynamics simulation of p53^Y220C^—drug complexes

To account for the stability of the interacting protein and ligand in a dynamic aqueous environment and study the resulting structural changes, the docked complexes were subjected to molecular dynamics simulations. All the molecular dynamics (MD) simulations were performed in Desmond using OPLS3e force field [46]. The mutant and stable-variants of p53 protein and, protein-drug complexes were solvated in water boxes. TIP3P water model was used. NaCl was used to neutralize the system and an additional concentration of 0.15 M was added. After system generation, Brownian motion simulation was done for 100 ps to minimize the systems. The systems were relaxed using relaxation protocol in Maestro, in which the following series of simulations were performed: Brownian dynamics simulations for 100 ps at 10 K with restraints on solute heavy atoms; MD simulations in NVT ensemble at 10 K with restraints on solute heavy atoms for 12 ps using Langevin thermostat; simulation in NPT ensemble for 12 ps with restraints for solute heavy atoms at 10 K at 1 atm pressure using Langevin thermostat and Langevin barostat; simulation in NPT ensemble for 12 ps with restraints for solute heavy atoms at 300 K and 1 atm pressure using Langevin thermostat and Langevin barostat; and simulation in NPT ensemble for 24 ps at 300 K and 1 atm pressure using the same thermostat and barostat [46]. After relaxing the system, production MD simulations were performed in NPT ensemble with a timestep of 2 fs. Restraints were not applied to any atoms. Nose–Hoover chain method was used to maintain temperature at 310 K, and pressure was maintained at 1 atm using Martyna-Tobias-Klein barostat [46]. For each system, simulation was performed for 200 ns and the data from last 100 ns were used for all the analyses.

### Energy calculations

All the energy calculations were done using Prime module in Maestro [46]. Prime performs single-point molecular mechanics energy calculation using OPLS3e force field and VSGB 2.0 energy model [46]. The protein–ligand binding free energies are computed using Prime MM-GBSA.

### Cell lines and reagents

Human isogenic hepatocarcinoma HuH-6 (representative of wild type p53) and HuH-7 (harboring Y220C p53 mutation) cells were purchased from the Japanese Collection of Research Bioresources (JCRB Cell Bank, Tokyo, Japan). Cells were cultured in Dulbecco’s modified Eagle’s medium (DMEM; Gibco BRL, Grand Island, NY, USA) supplemented with 5% fetal bovine serum (Fujifilm WAKO Pure Chemical Corporation, Osaka, Japan) and 1% antibiotics (penicillin–streptomycin) in 5% CO_2_ and 95% air humidified incubator.

### Cell viability assay

Cells were seeded into 96-well plate (5000 cells/well) and incubated for 24 h. The following day, CAPE (5–30 μM) was added to the culture medium for next 24 h followed by cell viability assay using MTT (3-(4,5-Dimethylthiazol-2-yl)-2,5-Diphenyltetrazolium Bromide) (Sigma Aldrich, Tokyo, Japan) following the manufacturer’s instructions. Control and CAPE treated cells were incubated with MTT (0.5 mg/ml) at 37 °C, 5% CO_2_ for 4 h. MTT-containing medium was replaced with DMSO (100 μl) in each well. Absorbance of the blue chromogen was measured at 570 nm using a spectrophotometer (Tecan Group Ltd., Männedorf, Switzerland).

### Luciferase reporter assays

PG13-Luc plasmid (bearing 13 repeats of the p53WT binding sequence) was a kind gift from Professor Bert Vogelstein. pWWP-Luc carrying the full p21^WAF-1^ promoter was purchased from AddGene, MA USA. The plasmids were transiently transfected into HuH-6 and HuH-7 cells using X-tremeGENE 9 HP DNA (Roche, Basel, Switzerland), following the manufacturer's protocol. In brief, cells were plated into a 6-well plate, and 3 μg of each plasmid was transfected into cells at a ratio of 3:1 of transfection reagent to DNA in antibiotic-free Opti-MEM (Invitrogen) media. The cells were cultured in serum-free media for 12–15 h after which serum-free media was replaced with complete growth media for the recovery of cells. After 24 h, some cells were taken to examine the transfection efficacy by firefly luciferase assay using untransfected cells as a control. Rest of the cell were seeded in a 6-well plate and allowed to adhere to substratum overnight. Next day, cells were treated with 10 μM and 30 μM CAPE for 24 h followed by preparation of lysates in passive lysis buffer. The luciferase activity was measured using firefly luciferase assay from Dual-Luciferase reporter assay system kit (Promega, WI, USA) and Infinite 200 PRO, luminescent plate reader (Tecan infinite M200® Pro) (Mannedorf, Switzerland) and was normalized/µg of the protein.

### Western blotting

Control and CAPE-treated cells were harvested after 24 h, lysed using RIPA Lysis Buffer (Thermo Fisher Scientific, Waltham, MA, USA) containing complete protease inhibitor cocktail (Roche Applied Science, Mannheim, Germany) and shaken in a cold room for 30 min. Lysates were centrifuged at 15,000 rpm for 15 min, and the supernatants were used for the Western-blotting analysis. The protein concentrations of whole-cell lysates were measured by the Pierce BCA Protein Assay Kit (Thermo Fisher Scientific, Waltham, MA, USA). The cell lysates (10–40 μg) were separated in 6–15% SDS-polyacrylamide gel electrophoresis (SDS-PAGE) and transferred to a polyvinyl dene difluoride (PVDF) membrane (Millipore, Billerica, MA, USA) using a semi-dry transfer blotter (ATTO Corporation, Tokyo, Japan). Membranes were blocked with 3% fraction-V bovine serum albumin at room temperature for 2 h. Blocked membranes were probed with the following target protein-specific primary antibodies: p53 (DO-1), BAX (H20X) (Santa Cruz Biotechnology, Paso Robles, CA, USA]; Mortalin (376) [raised in our laboratory]; and p21^WAFI^ (12D1) (Cell Signaling Technology, Danvers, MA, USA] at 4 °C overnight. The blots were incubated with the following secondary antibodies conjugated to horseradish peroxidase: anti-rabbit IgG, anti-goat IgG, and anti-mouse IgG (Santa Cruz Biotechnology, CA, USA) and developed by enhanced chemiluminescence (ECL) (GE Healthcare, Buckinghamshire, UK). Anti-β-actin antibody (Abcam, Cambridge, UK) was used as an internal loading control. ImageJ (National Institutes of Health, Bethesda, MD, USA) software was used to quantitate the protein signals.

### Statistical analysis

The mean and standard deviation of data from three or more independent experiments were calculated. The degree of significance between the control and treated experimental samples was determined using an unpaired t-test (GraphPad Prism GraphPad Software, San Diego, CA, USA). Statistical significance was defined as non-significant (^ns^ *p* value < 0.05), significant (*p value ≤ 0.05), very significant (**p value ≤ 0.01), and highly significant (***p value ≤ 0.001).

## Results

Y220C is a common mutation observed in p53 across various cancer types [47]. The replacement of tyrosine by cysteine causes disruption in the hydrophobic core maintained by the benzene ring of tyrosine, creating a surface crevice [9]. This leads to thermodynamically unstable p53 structure. However, as mentioned, it is possible to restore the structural stability in these mutant proteins. Therefore, here we explored the potential of CAPE to rescue the mutant p53^Y220C^ by binding to its crevice.

### PK7242 and CAPE have a similar interaction pattern with p53^Y220C^

**Fig. 1:**
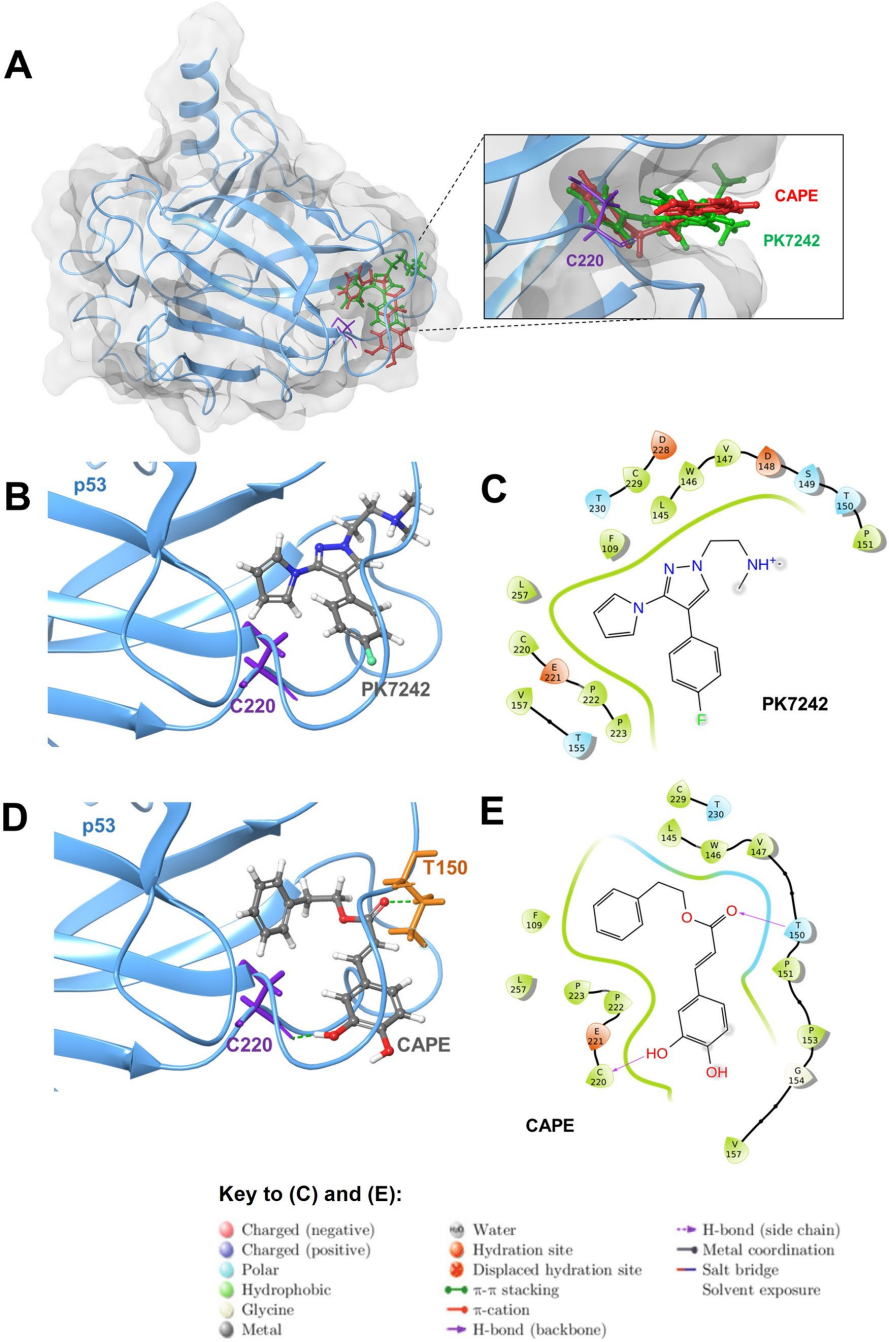
Binding of CAPE and PK7242 with p53^Y220C^. **(A)** Superimposed structures of CAPE (red) and PK7242 (green) bound at the mutation crevice of p53^Y220C^ molecule. The orientation of both the molecules at the docked site was found to be similar. Three-dimensional representation of PK7242 (**B**) and CAPE (**D**) at the mutation crevice of p53^Y220C^ molecule after docking. Molecular interaction of PK7242 (**C**) and CAPE (**E**) with p53^Y220C^

The crystallized complex of p53^Y220C^ mutant with the small molecule PK7242 bound to its mutation crevice was taken as a reference system for molecular modelling studies [44]. Molecular analysis was done to analyze the docking of CAPE in PK7242-binding site/mutation crevice of p53^Y220C^. Firstly, PK7242 was re-docked into the mutation crevice of p53^Y220C^, and the binding pose obtained was compared with pose in the crystal structure to check for the closeness. The pyrrole moiety of PK7242 in both the cases was buried in the protein cavity and the pyrazole ring was sandwiched between the loops bordering the cavity (Supplementary Fig. 1). The molecular interaction pattern was also the same. The docking score thus obtained served as a reference for comparing CAPE binding, and it also validated the docking method used in the study. We found that similar to PK7242, CAPE also docked well within the p53^Y220C^ mutation crevice (Fig. 1A). Interestingly, docking score of CAPE was higher as compared to that of PK7242 (Table 1). As shown in Fig. 1B, the docking pose of PK7242 was similar to its pose in the crystal structure (PDB 3ZME) [44], and the interactions were mostly hydrophobic (as shown in the two-dimensional interaction plot in Fig. 1C). CAPE formed two hydrogen bonds with the residues in the crevice (C220 and T150) of the p53^Y220C^ along with other hydrophobic interactions (Fig. 1D and E). Based on the docking score and molecular interactions, CAPE was found to be a better fit for the crevice of p53^Y220C^ as compared to PK7242.

**Table 1:** Docking scores and calculated free energies of binding of CAPE and PK7242 at p53^Y220C^ mutation crevice (the errors indicate the standard error of the mean)

	CAPE	PK7242
Docking score	− 9.115	− 6.611
Free energy of binding (kcal/mol)	− 60.31 ± 0.10	− 43.44 ± 0.09

**Fig. 2:**
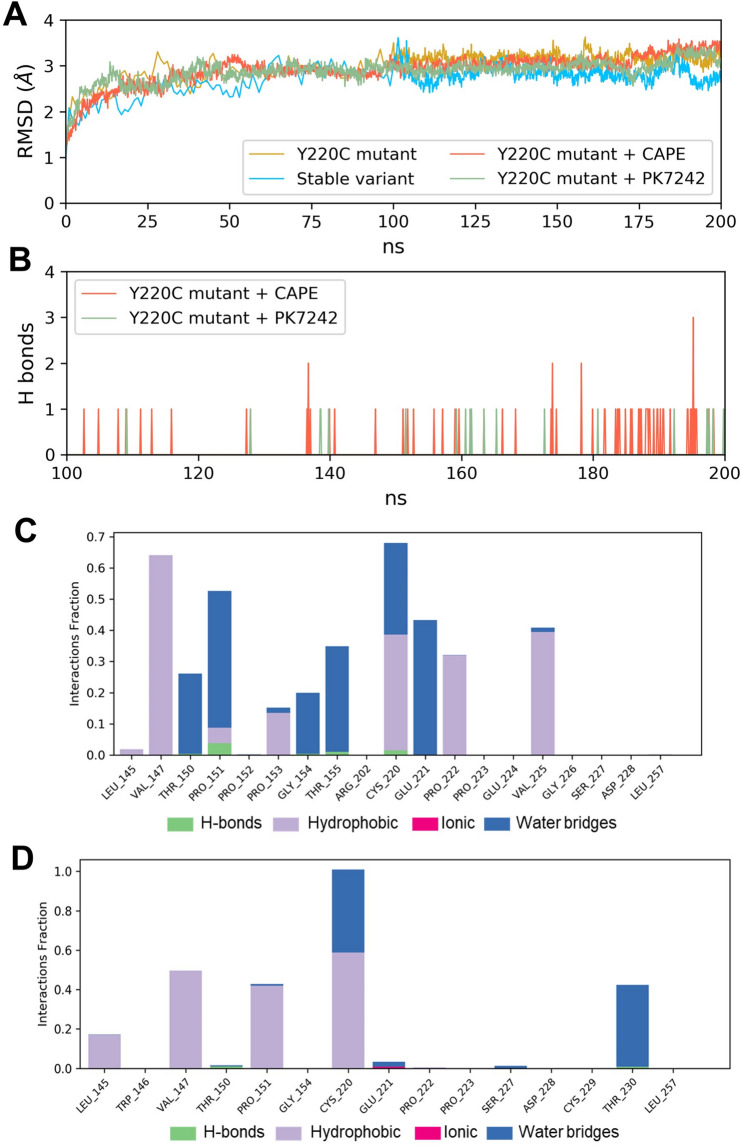
Analysis of molecular dynamics simulation trajectories. **(A)** Root mean square deviations of heavy atoms in the simulated structures with respect to the starting structure over 200 ns of simulation run. **(B)** Number of hydrogen bonds formed between the ligands and p53^Y220C^ crevice during the last 100 ns of each simulation. Interactions fraction diagram of CAPE (**C**) and PK7242 (**D**) at the mutation crevice of p53^Y220C^. The fraction of simulation time during which hydrogen bonds, hydrophobic contacts, ionic contacts, and water bridges are retained with different residues at the mutation crevice of p53^Y220C^ is shown in these plots

To further confirm the stability of these observed molecular interactions we used molecular dynamics simulations (MDS). MDS was performed for four different systems: p53 stable-variant (wild type representative), p53^Y220C^, p53^Y220C^ + CAPE and p53^Y220C^ + PK7242. The simulated structures in all the four cases were stable as reflected by the root mean square deviation (RMSD) calculated for heavy atoms of protein and ligand with respect to the initial structure throughout the 200 ns time scale of simulations (Fig. 2A). RMSD converged and hence we used the simulation data from last 100 ns for detailed analysis.

The number of hydrogen bonds between the small molecules—CAPE and PK7242 and mutant p53 during the course of simulation is shown in Fig. 2B. Since there were no hydrogen bonds between the ligands and the protein for substantial duration in the simulation trajectory, we can suggest that the hydrophobic interactions play major role in stabilizing the CAPE-p53^Y220C^ interactions. Throughout the simulation, the hydrophobic phenethyl group of CAPE was found buried inside the hydrophobic core of the crevice, while its hydrophilic caffeic acid group interacted with the protein residues surrounding the crevice and water molecules. Figure 2C shows the interactions of CAPE with different residues in the crevice of p53^Y220C^ during the simulation. The term ‘interactions fraction’ indicates the fraction of simulation time (100 ns) for which interaction of ligand with a specific residue of protein was maintained. Majority of the interactions of CAPE with the protein were found to be through hydrophobic contacts and water bridges, which differs from that of PK7242 where the water bridges were relatively less dominant/abundant suggesting that water molecules might also play an important role in mediating the CAPE-p53 interactions.

### CAPE binding provided stability to p53^Y220C^ structure

**Fig. 3:**
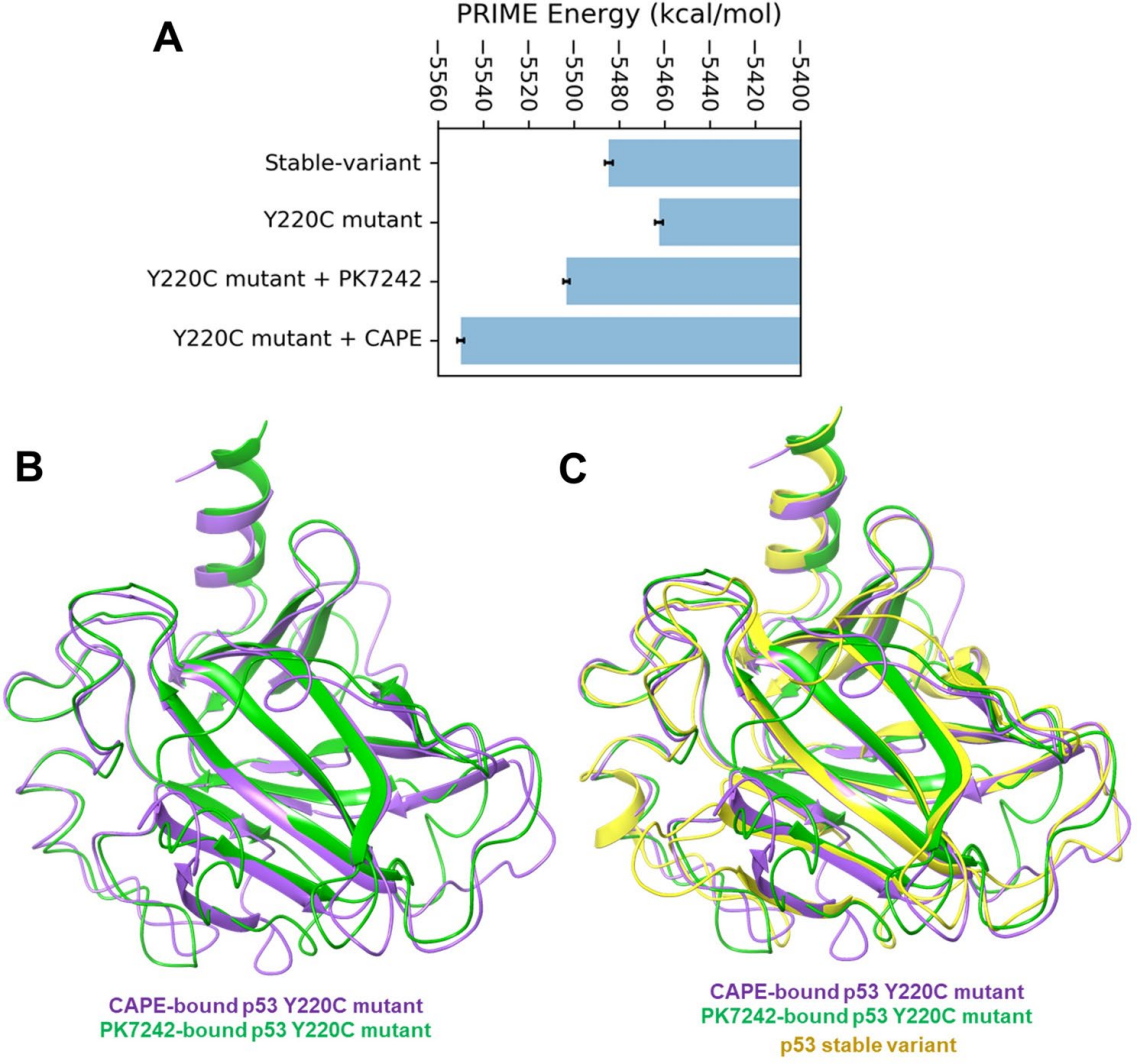
PRIME energies and structural integrity of the simulated complexes. **(A)** PRIME energies of p53 stable-variant, p53^Y220C^, p53^Y220C^ + CAPE complex and p53^Y220C^ + PK7242 complex during last 100 ns of simulation. CAPE bound to p53^Y220C^ had the lowest PRIME energy. The error bars indicate standard error of the mean. **(B)** Structure of CAPE-bound p53^Y220C^ (purple) superimposed with structure of PK7242-bound p53^Y220C^ (green). **(C)** Structures of CAPE-bound p53^Y220C^ (purple) and PK7242-bound p53^Y220C^ (green) superimposed with structure of the p53 stable-variant (yellow). The RMSD of the superimposed structures did not indicate any significant structural alterations in the complexes after binding with ligands and subsequent simulations

We next performed binding energy analysis of  CAPE and PK7242 with p53^Y220C^ using the last 100 ns of simulation trajectories. It was found that magnitude of binding free energy was higher for CAPE compared to that of PK7242 (Table 1). Hence, it was predicted that the CAPE has higher binding affinity for crevice of p53^Y220C^. Further, we also examined the PRIME energies of the optimized free wild type and mutated protein structure and the protein–ligand complexes (p53-stable variant, p53^Y220C^ without any ligand, p53^Y220C^ with PK7242/CAPE, respectively) (Fig. 3A). These relative energy terms allowed comparison of free and ligand bound states of the protein to analyze favorable energetics. Among all the four states, energy of p53^Y220C^ + CAPE complex was the least suggesting that CAPE binding offers better stability to p53^Y220C^ as compared to PK7242.

To study the structural changes in p53^Y220C^ due to CAPE binding, the drug bound complexes were compared with p53-stable variant. The zinc ion held on to the p53 molecule in all the simulated systems. A representative structure was chosen from each of the simulation trajectory, and these structures were superimposed using Maestro suite [46]. After superimposition by the heavy atoms of protein backbone, RMSD between the structures were calculated. RMSD between superimposed p53^Y220C^ + CAPE complex and p53^Y220C^ + PK7242 complex was just 2.96 Å indicating that structural alterations induced by CAPE binding were not very different compared to that of PK7242 binding (Fig. 3B). RMSD of p53-stable variant structure superimposed with p53^Y220C^ + CAPE and p53^Y220C^ + PK7242 complexes were 2.75 Å and 3.18 Å, respectively (Fig. 3C). Lower RMSD of p53^Y220C^ + CAPE complex with respect to p53-stable variant also confirmed the overall structural integrity of the CAPE bound mutant.

### Experimental evidence of wild-type like activity in p53^Y220C^ upon CAPE treatment

**Fig. 4:**
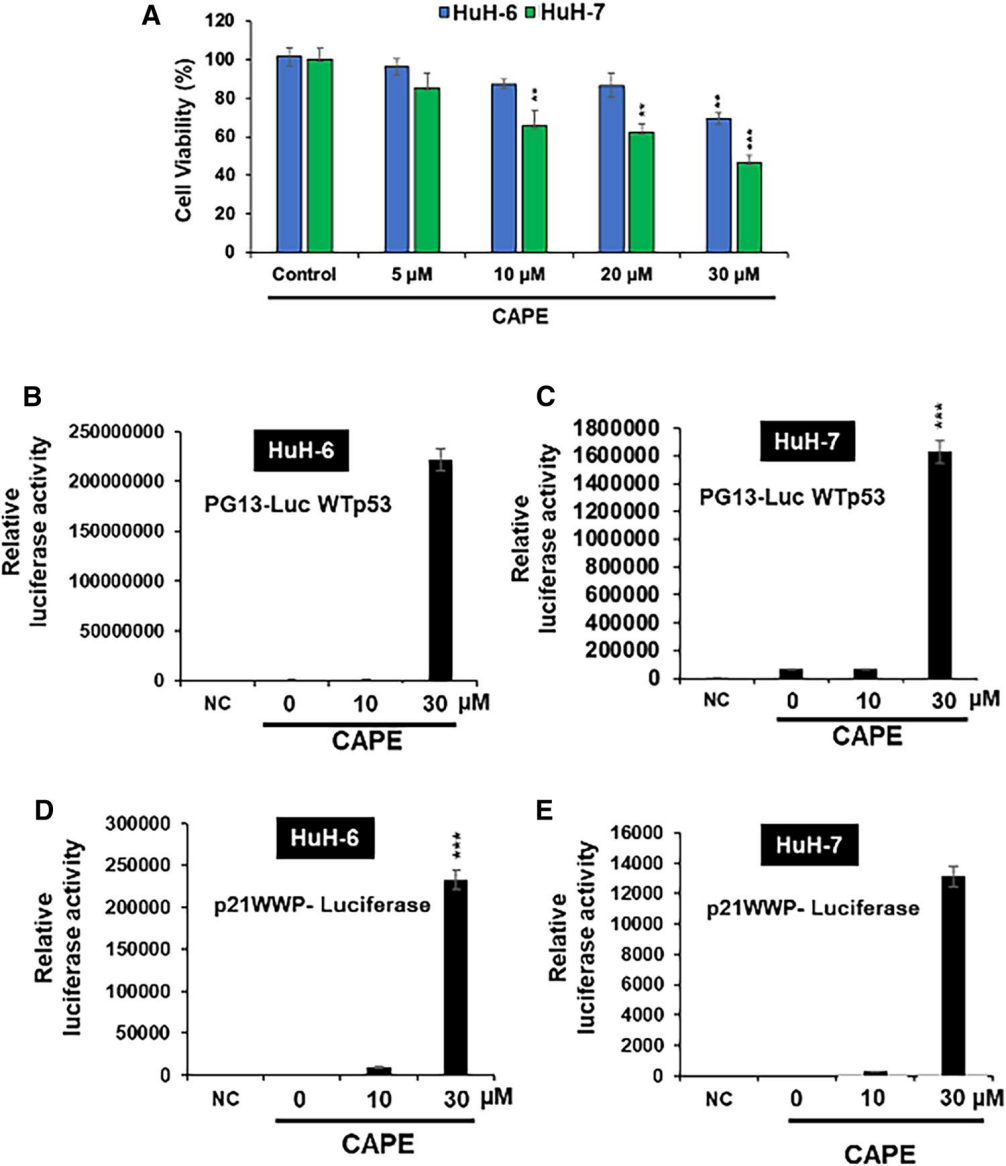
Activation of wild type like p53 activity in p53 mutant cell line. **(A)** Dose dependent cytotoxicity of CAPE in HuH-6 and HuH-7 cells. **(B–E)** Detection of p53^wt^-dependent luciferase activity by reporter plasmids containing the p53-binding synthetic sequence (PG-13Luc) (**B**, **C**) of p21^WAF−1^ promoter (WWP-Luc) (**D**, **E**). Increase in p53^wt^-dependent luciferase activity was detected in CAPE treated HuH-6 cells (**B**, **D**) and HuH-7 (**C**, **E**) cells

Next, we set out to experimentally validate the predictions by studying the effect of CAPE on cells expressing p53^wt^ (HuH-6) and p53^Y220C^ (HuH-7) proteins. As shown in Fig. 4A both HuH-6 and HuH-7 cells showed cytotoxicity of CAPE. Of note, HuH-7 cells containing p53^Y220C^ showed stronger cytotoxicity in comparison to HuH-6 cells containing p53^wt^ in the range of 5 to 30 μM CAPE treatments. Based on the cytotoxicity profile, we chose 10 and 30 μM dose of CAPE for further experiments.

In light of the information that p53^Y220C^ is structurally unstable and transcriptionally inactive due to lack of its binding to DNA, we subjected HuH-6 and HuH-7 control and CAPE-treated cells to p53^wt^-driven luciferase reporter assays. As expected, p53^wt^-driven luciferase reporter activity was enhanced in CAPE treated HuH-6 cells (Fig. 4B). Interestingly, increase in p53^wt^-driven luciferase reporter activity was also observed in CAPE-treated HuH-7 cells (Fig. 4C). In order to further investigate whether CAPE-mediated reactivation of p53^wt^-function in HuH-7 cells could effectively initiate its downstream signaling pathways, we next carried out p21^WAF−1^ promoter-dependent luciferase reporter assay. As shown in Fig. 4D and E, both HuH-6 and HuH-7 cells showed remarkable increase in p21-promoter dependent luciferase reporter activity in CAPE (30 μM) treated cells.

**Fig. 5:**
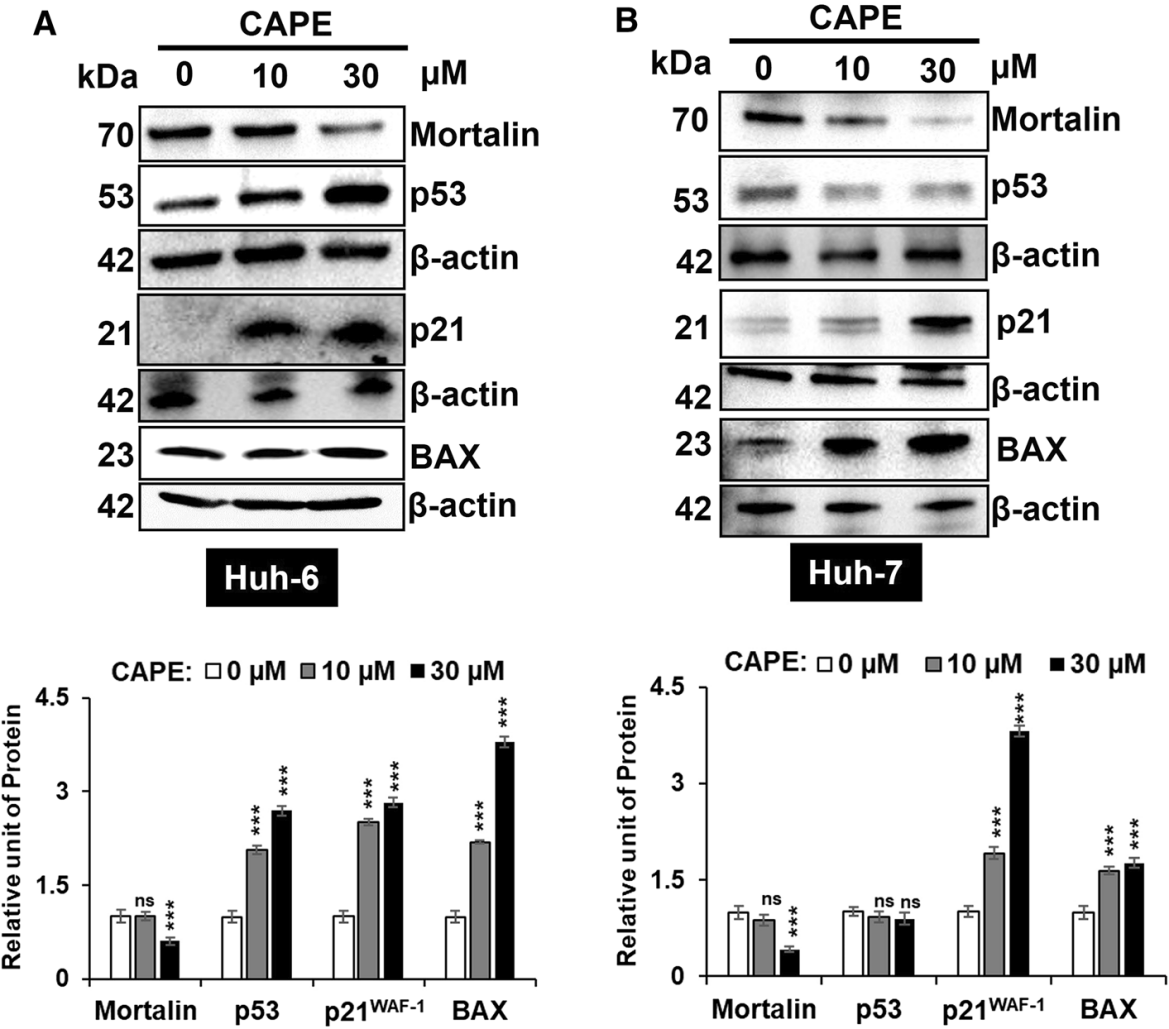
Expression of p53^wt^-downstream effector proteins in CAPE treated HuH-6 and HuH-7 cells. **(A)** CAPE-treated HuH-6 cells showed dose-dependent decrease in mortalin and increase in p53, p21^WAF−1^ and BAX proteins. **(B)** CAPE-treated HuH-7 cells showed dose-dependent decrease in mortalin and increase in p21^WAF−1^ and BAX proteins. Of note, p53^Y220C^ did not show increase in expression. However, has restored p53^wt^ function as evidenced by increase in p21^WAF−1^ and BAX, and reporter assays shown in Fig. 4

We also performed Western blotting for proteins involved in p53^wt^-driven control of cell cycle and growth arrest in control and CAPE treated HuH-6 and HuH-7 cells. It is well established that mortalin, a heat shock protein enriched in cancer cell, sequestrates p53 in the cytoplasm, restricting its translocation to nucleus and hence the transcriptional activation function [48]. It was previously shown that CAPE abrogates mortalin- p53 interaction causing its translocation and reactivation of transcriptional activation function and growth arrest/apoptosis of cells mediated by specific downstream effectors [38]. In view of this, we examined the expression of mortalin and p53 in control and CAPE treated HuH-6 and HuH-7 cells. As shown in Fig. 5A and B, the expression of mortalin decreased in a dose-dependent manner in both the cell lines and was consistent with earlier reports [38, 43]. Of note, whereas HuH-6 cells showed 2–3 folds increase in p53^wt^ protein, HuH-7 cells did not show any significant change in p53 protein level in several independent experiments. In order to assess p53^wt^ function, we investigated the expression of p21^WAF−1^ and BAX, downstream genes in p53 pathway involved in cell cycle arrest and apoptosis respectively. In line with the reactivation of transcriptional-activation function of p53^wt^ in CAPE treated cells, p21^WAF−1^ and BAX proteins showed upregulation in the treated HuH-6 cells. Of note, remarkable increase in expression of these proteins were observed in HuH-7 cells demonstrating restoration of p53^wt^ function in p53^Y220C^ cells by CAPE.

## Discussion

CAPE has earlier been shown to possess anticancer potential for a variety of cancer cell types. It caused activation of DNA damage response, upregulation of GADD45 and reactivation of tumor suppressor p53 in cancer cells [38, 43]. The p53 protein is the most mutated protein across various cancer types. The loss-of function mutations in p53 either directly disrupt its interaction with DNA or make the structure unstable effecting it transcriptional activation function. Hence, there is a need to check the response of p53 targeting drugs on these mutant variants to account for wider applicability. We investigated the potential of CAPE for its effect on p53^Y220C^ mutant. The higher affinity of CAPE to p53^Y220C^ crevice as compared to the known co-crystallized binder and restoration small molecule-PK7242 (as evident through the binding free energy calculations) indicated that p53^Y220C^ could be targeted by CAPE in cancer cells. Molecular dynamics simulations analyses showed that CAPE-p53^Y220C^ interactions were stable. As the solvation of the crevice in p53^Y220C^ has been attributed to the destabilization of the p53 molecule, the association of hydrophobic phenethyl group of CAPE with the crevice prevented its solvation and thereby stabilized the molecule. The degree of stabilization was found to be higher in case of CAPE, compared to PK7242, as suggested by PRIME energy calculations. RMSD calculations after superimposing the equilibrated structures of CAPE-bound p53^Y220C^ with the stable-variant did not show any significant alterations in the core structure of the p53 protein. With these in silico predictions, we performed cell-based experiments using human hepatocarcinoma possessing isogenic p53^wt^ (HuH-6) and p53^Y220C^ (HuH-7) harboring cells. CAPE treatment (5–30 μM) showed stronger effect on HuH-7 cells. Wild type p53-dependent synthetic (PG-13Luc) and p21-promoter (pWWP-Luc) reporter assays revealed increase in reporter activity both in HuH-6 and HuH-7 cells. Reactivation of wild type p53 (HuH-6) was in line with other cancer cell types (MCF7, U2OS) [38]. Furthermore, wild type p53 activity in HuH-7 cells supported the bioinformatics analysis. CAPE-treated cells also showed increase in expression of p21^WAF−1^ and BAX proteins that are involved in p53^wt^-induced growth arrest or apoptosis, respectively. These data demonstrated that CAPE caused restoration of p53^wt^ in HuH-7 cells that possess p53^Y220C^.

## Conclusion

In this study we explored the potential of CAPE to restore the function of structurally unstable p53^Y220C^. Molecular docking, molecular dynamics simulations and free energy calculations revealed that CAPE could bind to the mutation crevice in p53^Y220C^ and re-established the hydrophobic core disrupted by the mutation. Cell-based molecular assays further demonstrated reactivation of p53^wt^ function yielding growth arrest/apoptosis in p53^Y220C^ harboring cells. CAPE is suggested as a potential natural small molecule drug for treatment of p53^Y220C^ harboring cancers.

## Supplementary Information

Below is the link to the electronic supplementary material.

Supplementary file1 (DOCX 1304 KB)

## Data Availability

All data generated or analyzed during this study are included in this published article.
