# Supplementary material for: Caffeic acid phenethyl ester (CAPE) confers wild type p53 function in p53Y220C mutant: bioinformatics and experimental evidence
Source: Discov Oncol. 2021 Dec 20;12:64. doi: 10.1007/s12672-021-00461-2 (PMC8777538; doi:10.1007/s12672-021-00461-2)

**Caffeic acid phenethyl ester (CAPE) confers wild type p53 function**

**in p53^Y220C^ mutant: bioinformatics and experimental evidence**

Navaneethan Radhakrishnan^1$^, Jaspreet Kaur Dhanjal^2,3$^, Anissa Nofita Sari^2^, Yoshiyuki Ishida^4^,

Keiji Terao^4^, Sunil C. Kaul^2^, Durai Sundar^1*^ and Renu Wadhwa^2*^

**Supplementary information**

**Supplementary Figure 1. Redocking of PK7242 to p53^Y220C^.** Superimposed structures of redocked PK7242 (green) and PK7242 in crystal structure (red) bound at the mutation crevice of p53^Y220C^ molecule. The orientation of redocked PK7242 at the mutation crevice was found to be similar to that of crystal structure.


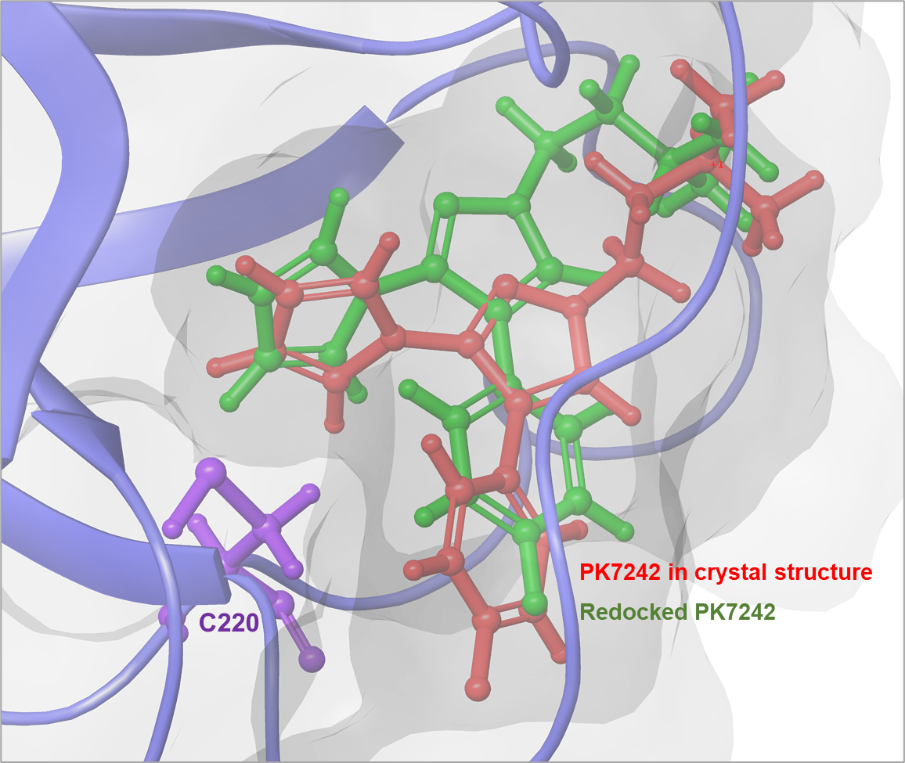


**Supplementary Figure 2:** The original uncropped western blot images reported in the main Fig. 5


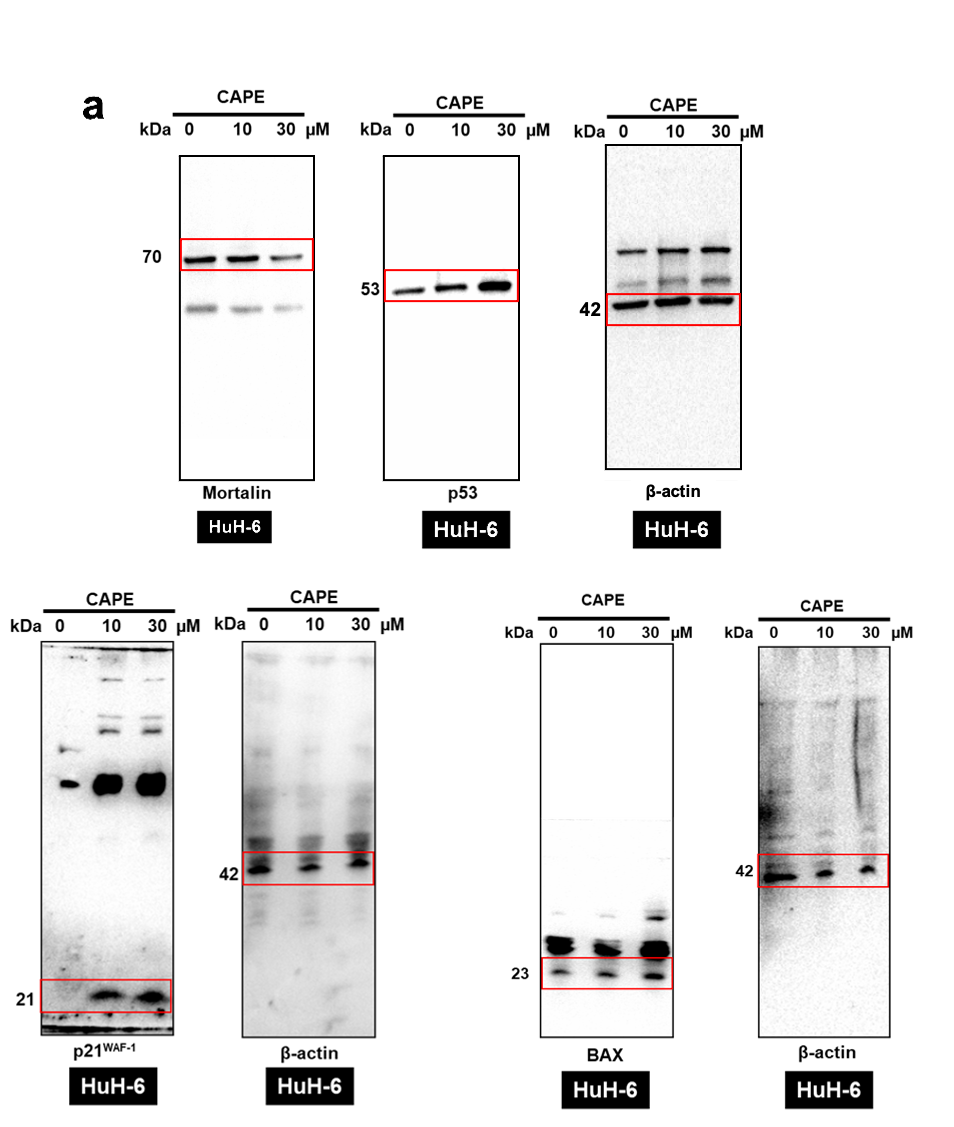


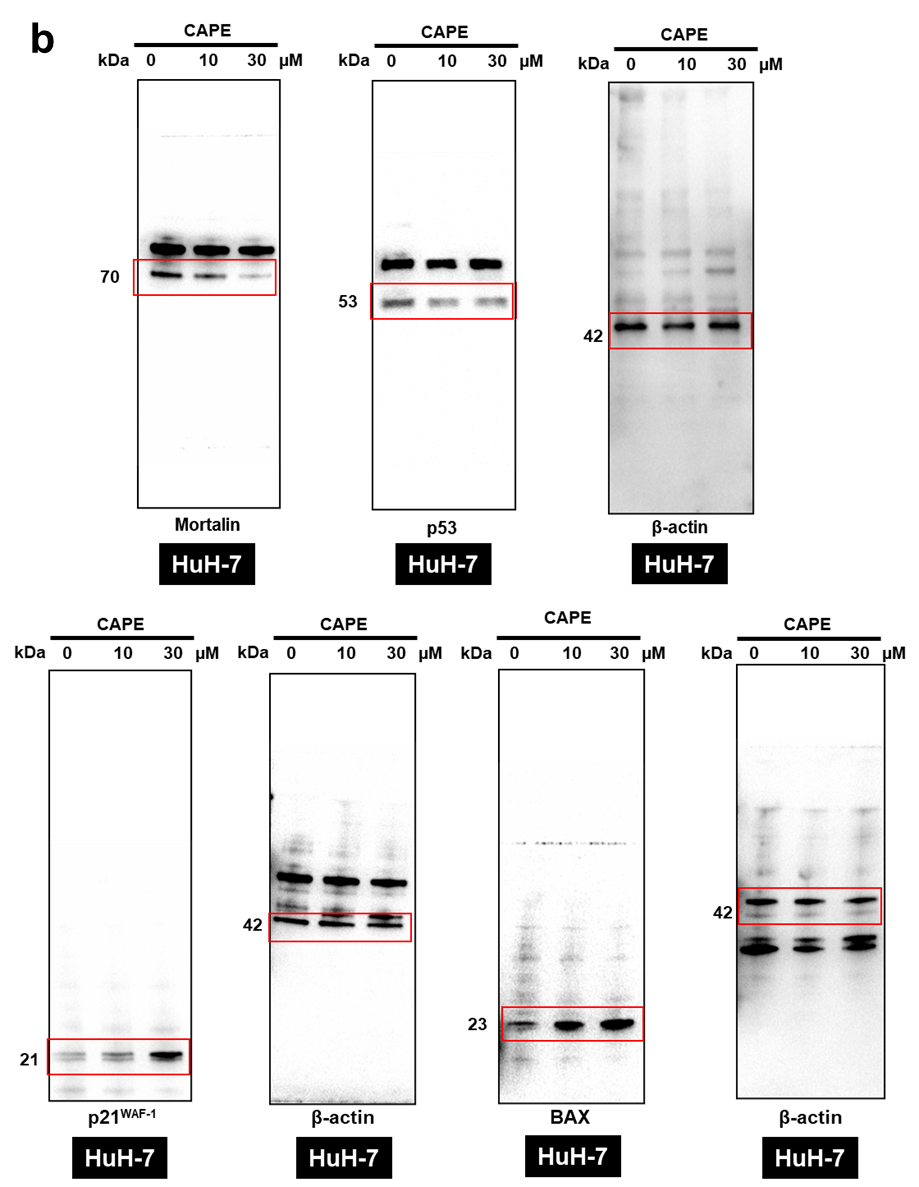

Supplement: Supplementary file 1 — Supplementary file1 (DOCX 1304 KB) [file 12672_2021_461_MOESM1_ESM.docx]
